# Electrochemical Detection of Single-Nucleotide Polymorphism Associated with Rifampicin Resistance in *Mycobacterium tuberculosis* Using Solid-Phase Primer Elongation with Ferrocene-Linked Redox-Labeled Nucleotides

**DOI:** 10.1021/acssensors.1c01710

**Published:** 2021-11-19

**Authors:** Mayreli Ortiz, Miriam Jauset-Rubio, Vasso Skouridou, Diana Machado, Miguel Viveiros, Taane G. Clark, Anna Simonova, David Kodr, Michal Hocek, Ciara K. O’Sullivan

**Affiliations:** † Departament d’Enginyeria Química, Universitat Rovira i Virgili, Avinguda Països Catalans 26, 43007 Tarragona, Spain; ‡ Global Health and Tropical Medicine, GHTM, Instituto de Higiene e Medicina Tropical, IHMT, Universidade Nova de Lisboa, Rua da Junqueira, 100, 1349-008 Lisbon, Portugal; § Faculty of Infectious and Tropical Diseases, London School of Hygiene & Tropical Medicine, WC1E 7HT London, U.K.; ∥ Institute of Organic Chemistry and Biochemistry, Czech Academy of Sciences, Flemingovo nam.2, 16610 Prague 6, Czech Republic; ⊥ Department of Organic Chemistry, Faculty of Science, Charles University, Hlavova 8, 12843 Prague 2, Czech Republic; # Institució Catalana de Recerca i Estudis Avançats, Passeig Lluis Companys 23, 08010 Barcelona, Spain

**Keywords:** single-point mutation, single-nucleotide polymorphism (SNP), ferrocene-labeled nucleotides, nucleoside triphosphates, solid-phase primer elongation, Klenow (exo-) DNA polymerase

## Abstract

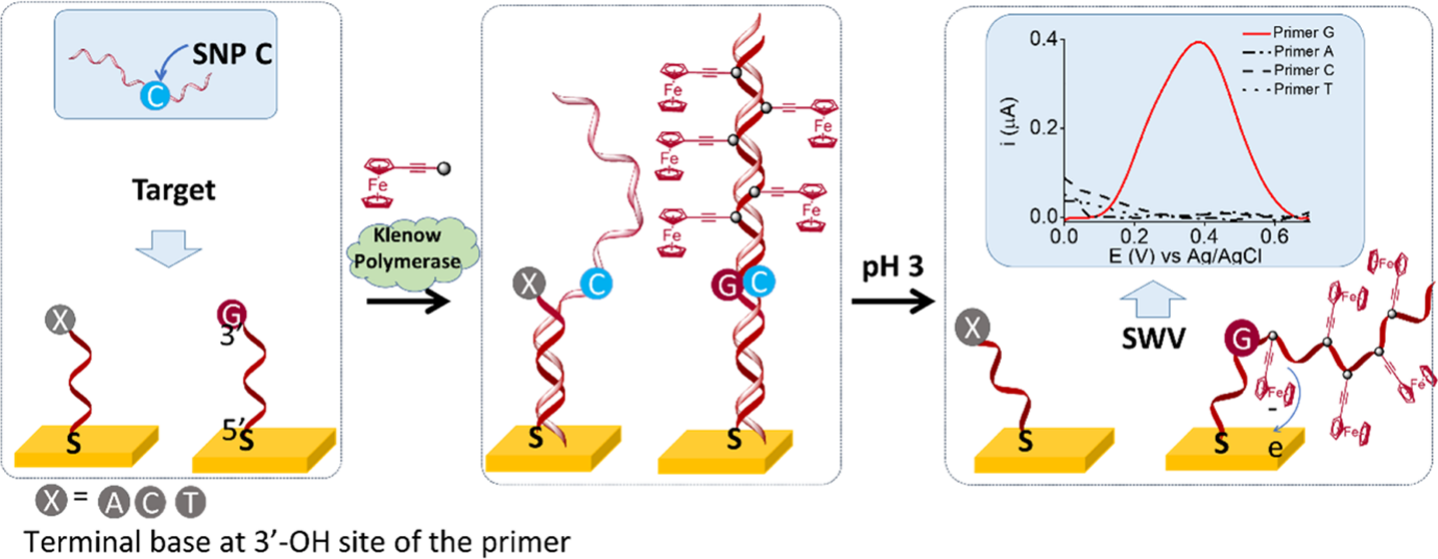

Here, we report the electrochemical detection of single-point mutations using solid-phase isothermal primer elongation with redox-labeled oligonucleotides. A single-base mutation associated with resistance to rifampicin, an antibiotic commonly used for the treatment of *Mycobacterium tuberculosis*, was used as a model system to demonstrate a proof-of-concept of the approach. Four 5′-thiolated primers, designed to be complementary with the same fragment of the target sequence and differing only in the last base, addressing the polymorphic site, were self-assembled via chemisorption on individual gold electrodes of an array. Following hybridization with single-stranded DNA, Klenow (exo-) DNA polymerase-mediated primer extension with ferrocene-labeled 2′-deoxyribonucleoside triphosphates (dN^Fc^TPs) was only observed to proceed at the electrode where there was full complementarity between the surface-tethered probe and the target DNA being interrogated. We tested all four ferrocenylethynyl-linked dNTPs and optimized the ratio of labeled/natural nucleotides to achieve maximum sensitivity. Following a 20 min hybridization step, Klenow (exo-) DNA polymerase-mediated primer elongation at 37 °C for 5 min was optimal for the enzymatic incorporation of a ferrocene-labeled nucleotide, achieving unequivocal electrochemical detection of a single-point mutation in 14 samples of genomic DNA extracted from *Mycobacterium tuberculosis* strains. The approach is rapid, cost-effective, facile, and can be extended to multiplexed electrochemical single-point mutation genotyping.

As a deeper understanding of the heterogeneous nature of the human disease is elucidated, there has been an increasing emphasis on the importance of patient stratification and personalized medicine. To achieve this goal of a pharmacogenomic approach for the treatment of disease, cost-effective, easy-to-use, and rapid tools for point-of-care diagnostics are required. The advent of next-generation sequencing has not only revealed the presence of millions of single-nucleotide polymorphisms^1^ across the human genome but has also highlighted the role of these single-point mutations in bacterial resistance.^2^

This is of particular importance for effective triaging against infectious diseases, such as tuberculosis (TB). TB can be asymptomatic or latent, resulting in a delay in diagnosis, and, in 2019, an estimated 10 million people fell ill with the disease. This delay in diagnosis is compounded by the increasing prevalence of variants of *Mycobacterium tuberculosis* resistant to rifampicin, the first-line drug of choice for TB treatment.^3,4^ The sequencing of the whole genome of *Mycobacterium tuberculosis*(5) revealed that 95–98% of all rifampicin-resistant strains is related to single-nucleotide polymorphisms (SNPs), located in the 81 bp RIF-resistance determining region (RRDR) of the beta subunit of the RNA polymerase (rpoB) gene.^6−11^ The World Health Organization reported that around half a million people developed rifampicin-resistant TB in 2019,^12^ and in 2010, recommended the use of the Xpert MTB/RIF assay (Cepheid, Sunnyvale, CA) for the detection of TB and rifampicin resistance, and while it is widely used, its implementation in developing countries is hampered by many issues, including cost, maintenance, and power requirements.^13−15^

There is thus still a mature need for reliable, cost-effective, and rapid tests for the detection of the clinically relevant mutations/SNPs conferring rifampicin resistance in *Mycobacterium tuberculosis*.^10^

A plethora of SNP detection methods have been reported, with the majority requiring a nucleic acid amplification step including PCR/qPCR,^8,16−18^ solid-phase,^19−21^ or isothermal amplification.^22−24^ The approach used in the Sanger sequencing method^25^ has been extrapolated for the detection of single nucleotides via the single-base extension (SBE) of primers,^26^ which was then applied to the detection of genetic diseases^27^ and point mutations^28^ and since then has been extensively employed for the detection of single-point mutations as well as is being used in solid-phase array platforms, such as the reversible terminator next-generation sequencing approach employed by Illumina.^29^ Solid-phase minisequencing for the detection of single-nucleotide variations was first reported in 1990,^30^ and then extended to the detection of mutations^31^ and polymorphisms using array-based primer extension, termed APEX.^32,33^ In APEX, single-stranded DNA (ssDNA) generated from PCR products are hybridized to primers surface-tethered on a microarray chip. The primers are immobilized via their 5′ ends, exposing the 3′-OH of the terminal oligonucleotide of the primer, which is specifically designed to hybridize the ssDNA one single base downstream of the SNP being interrogated. Following hybridization and incorporation of fluorescently labeled 2′,3′-dideoxyribonucleotides, any further elongation/extension of the immobilized primer is terminated due to the lack of 3-′OH on the ddNTPs and the SNP identified via the fluorescent label. CCD imaging of a microarray functionalized with diverse primers allows simultaneous, multiplexed detection and identification of SNPs.^34^

Electrochemical detection of single-base/nucleotide extension using redox-labeled 2′,3′-dideoxyribonucleotides has been reported as an alternative to fluorescence. Ferrocene is a widely accepted electrochemical marker due to its unequivocal signal in the potential window compatible with the majority of biomolecules.^35^ In the first report by Brazill and Kuhr,^36^ a primer was labeled at the 5′ with ferrocene acetate, and following single-base extension, the authors demonstrated the ability to discriminate between extended and nonextended primers using capillary electrophoresis and sinusoidal voltammetric (CGE-SV) detection. In an effort to overcome the requirements for such stringent separation between an extended/unextended primer, the authors went on to carry out single-base extension using commercial dideoxy-ferrocene-acycloATP, ddFc-aATP (Motorola Life Sciences), with the product being detected both by CGE-SV and MALDI-TOF.^37,38^

We recently reported the preparation and incorporation of 2′,3′-dideoxyribonucleotides labeled with ferrocene, methylene blue, anthraquinone, and phenothiazine^39^ using solid-phase single-base extension. Gold electrodes of an array were functionalized via chemisorption with thiolated primers designed to hybridize to ssDNA targets one base downstream of the SNP to be interrogated. Following hybridization, a mixture of the redox-labeled ddNTPs were added and the surface-tethered primer extended, with subsequent voltammetric detection of the incorporated labeled 2′,3′-dideoxyribonucleotides. In further work, to reduce the background signal due to electrostatic interaction with methylene blue, this redox label was replaced with polyoxometalate labels.^40^ In both approaches, excellent discrimination between incorporated and nonincorporated ddNTPs was achieved, allowing unequivocal identification of the SNP under interrogation.

However, the synthesis and purification of redox-labeled ddNTPs are expensive, laborious, and quite complex, and the use of redox-labeled dNTPs presents a more straightforward approach. We have recently reported on the electrochemical detection of the ratiometric incorporation of four differentiable redox-labeled nucleotides, demonstrating their use in primer extension assays.^41^ To use labeled dNTPs rather than ddNTPs, we explored the possibility of solid-phase primer elongation for the detection of SNPs. In this approach, first reported in 2001, primers containing a variable base (A, G, T, C) at their 3′ end were covalently immobilized via their 5′ ends to glass slides. Single-stranded DNA containing the SNP to be interrogated was generated from PCR amplicons and allowed to hybridize to all four immobilized probes. Subsequently, solid-phase amplification was carried out using a Taq DNA polymerase and a mixture of dNTPs containing Cy3-dUTP. Primer elongation was only observed where there was full complementarity between the immobilized primer and ssDNA, and a fluorescent signal was measured at this spot of the glass slide, thus facilitating identification of the allele present at the SNP site under interrogation.^42^ This approach has since been reported for a variety of applications requiring the identification of SNPs,^43,44^ including whole-genome analysis,^45^ and new advances have been made to improve discrimination,^46^ as well as is being used for the ultrasensitive detection of specific DNA sequences.^47^

The objective of the work reported here was to develop an approach for the electrochemical detection of solid-phase primer elongation using ferrocene-labeled dNTPs for the unequivocal identification of SNPs. The motivation for this work was to develop a generic platform for the electrochemical detection of SNPs, which could be exploited in a portable device for the multiplexed identification of SNPs at the point of need. To date, many of the multiplexed SNP microarray platforms exploit fluorescence detection with CCDs, which inherently require cooling and complex optics. We were motivated to develop an alternative to fluorescence detection via the use of electrochemical detection, compatible with handheld potentiostats such as those used in glucometers, thus facilitating portability and cost-effectiveness. Exploiting our previous knowledge in the development of biosensors and in the use of redox-labeled nucleotides,^41^ we wanted to combine this know-how to demonstrate a proof-of-concept for the cost-effective, rapid, and facile detection of SNPs in a platform that could easily be expanded to multiplexed detection with a plethora of niche applications.

## Experimental Section

### Reagents

All reagents were of analytical grade and used as received. All electrochemical solutions were prepared in ultrapure water (18 MΩ·cm) using a Simplicity Water Purification System (Millipore, France).

Sodium chloride (NaCl), potassium dihydrogen phosphate (KH_2_PO_4_), potassium chloride (KCl), and phosphate-buffered saline (PBS) were purchased from Fisher Scientific (Spain), while sulfuric acid (95–97%) was purchased from Scharlau (Spain) and hydrochloric acid (35% v/v) from Panreac. 10-(3,5-bis((6-Mercaptohexyl)oxy)phenyl)-3,6,9-trioxadecanol (DT1)^48^ was purchased from SensoPath Technologies (Bozeman, MT).

KAPA2G Robust DNA polymerase from KAPABIOSYSTEMS was purchased from Sigma-Aldrich (Spain). Klenow fragment (exo-) DNA polymerase, agarose, DNA Gel Loading Dye (6X), GeneRuler Low Range DNA Ladder, natural dNTPs, and nuclease-free water were purchased from Fisher Scientific (Spain). DNA Clean & Concentrator and Oligo Clean & Concentrator kits were purchased from Ecogen (Spain), while GelRed nucleic acid stain was purchased from Biogen Científica (Spain).

dA^Fc^TP and dU^Fc^TP,^49^ dG^Fc^TP^50^ as well as dC^Fc^TP^51^ were prepared as previously reported. The dA^Fc^TP and dC^Fc^TP are now commercially available from Jena Biosciences (www.jenabioscience.com). The HPLC purified oligonucleotides were purchased from Biomers.net (Germany) and used as received. The sequences used (from 5′ to 3′) are listed in Table S1 in the Supporting Information.

### Samples of Biological Origin and DNA Extraction

DNA was extracted from *Mycobacterium tuberculosis* strains belonging to the culture collection of the Laboratório de Micobactérias, Unidade de Microbiologia Médica, Instituto de Higiene e Medicina Tropical, Universidade NOVA de Lisboa (IHMT-NOVA). The strains selected for DNA extraction were isolated from Portuguese tuberculosis patients as part of the routine mycobacteriology laboratory services provided by Universidade NOVA de Lisboa (Lisboa, Portugal) to the local hospitals. Given the retrospective nature of the work involving only anonymized bacterial isolates, informed consent was not required for this study. *Mycobacterium tuberculosis* gDNA was purified and concentrated using the cetyltrimethylammonium bromide (CTAB) method.^52^

### Primer Elongation Reaction in Solution

The primer elongation reaction in solution was carried out using 0.2 mM each dNTP including dN^Fc^TP at different percentages for optimization experiments (described in Section 3 of the Supporting Information) and 30% of each of the four dN^Fc^TP for the final approach. For optimization experiments, the reaction mixture only contained 0.5 μM primer G (fully complementary with target), while for the final approach, an individual reaction for each of the four primers was prepared to evaluate the specificity of the assay. In all of the cases, 1× buffer of Klenow (exo-) DNA polymerase, 2 U of Klenow fragment (exo-) DNA polymerase, and 0.5 μM ssDNA template were used. A PCR product was used as a positive control of the elongation reaction. The reactions were carried out at 37 °C for 25 min followed by 10 min at 60 °C for enzyme deactivation.

### Gel Electrophoresis Analysis

Agarose gel (4% w/v) stained with GelRed nucleic acid stain was prepared in 1× Tris borate–EDTA buffer (TBE) at pH 8. A total of 5 μL of each DNA sample was mixed with 4 μL of DNA Gel Loading Dye (6×), loaded on the 4% agarose gel and then run in the same buffer at 100 mV for 30 min. After electrophoresis, the intensity of the bands was estimated using the Gel Analysis option of ImageJ software (National Institutes of Health) (http://imagej.nih.gov/ij/) as detailed in the Supporting Information.

### Electrochemical Measurements

All electrochemical measurements were carried out using an Autolab model PGSTAT 12 potentiostat/galvanostat controlled with General Purpose Electrochemical System (GPES) software (Eco Chemie B.V., The Netherlands). Electrode arrays of 16 individual 1 mm^2^ square gold working electrodes (thickness of 150 nm) with a common gold counter electrode and a Ag silver reference electrode were used (Figure S1a). The electrodes were fabricated at Fraunhofer ICT-IMM, Germany, using a photolithographic process as previously reported.^53^ After the cleaning process (described in Section 1 of the Supporting Information), cyclic voltammograms were recorded in 0.1 M H_2_SO_4_ for each of the 16 working electrodes, and the gold reduction peaks overlapped to verify the reproducibility of the working electrode areas (Figure S1b,c).^53^

Immediately after cleaning (see details in Section 1 of Supporting Information), each thiolated primer (5 μM) was mixed with 50 μM alkylthiol 22-(3,5-bis((6-mercaptohexyl)oxy)phenyl)-3,6,9,12,15,18,21-heptaoxanol and DT1 (1:10 final molar ratio) in 1 M KH_2_PO_4_, drop cast on the working electrodes, and incubated for 4 h at 37 °C inside a humidity chamber to avoid evaporation. DT1 was used as a coimmobilizer to provide lateral spacing between the immobilized primers to thus avoid any steric hindrance that would inhibit access of the target DNA for hybridization to the surface-tethered primers.^48^ The electrode array was washed under stirring for 15 min in 1 M KH_2_PO_4_, then gently rinsed with Milli Q water, and finally dried with nitrogen.

Square wave voltammetry (SWV) was used to detect the incorporation of the Fc-labeled dNTPs. This technique was chosen over differential pulse voltammetry (DPV) or cyclic voltammetry (CV) due to the increased sensitivity and measurement time, but CV and DPV could also be used. The measurements were performed at 22 °C in 10 mM Tris buffer containing 100 mM KCl, pH 7.4. SWVs were recorded from 0 to 0.7 V vs Ag using a pulse amplitude of 0.1 V, a step potential of 10 mV, and a frequency of 25 Hz. Cyclic voltammograms were recorded from 0 to 0.6 V vs Ag at different scan rates.

### Solid-Phase Primer Elongation Reactions

A microfluidic housing containing four channels was created per electrode array for further individual reactions. First, the four 5′-thiolated primers + alkylthiol 22-(3,5-bis((6-mercaptohexyl)oxy)phenyl)-3,6,9,12,15,18,21-heptaoxanol were coimmobilized on each of the four individual electrodes of each channel as described above, followed by addition of a range of concentrations of 15 μL of ssDNA in 10 mM Tris buffer + 500 mM NaCl, pH 7.4, and left to hybridize for 20 min at 37 °C in a humidity chamber. The electrodes were then gently washed with the same buffer, and 15 μL of the reaction mixture (200 μM of total dNTPs (containing dN^Fc^TPs and native dNTPs), 1× buffer of Klenow (exo-) DNA polymerase buffer, and 2 U of Klenow fragment (exo-) DNA polymerase) was added to each channel and left to react for 5 min at 37 °C. The electrodes were then incubated for 5 min with glycine pH 3. The electrodes were finally washed twice, again with Tris buffer, the channel was filled, and the electrochemical measurements were carried out using SWV.

## Results and Discussion

**Figure 1:**
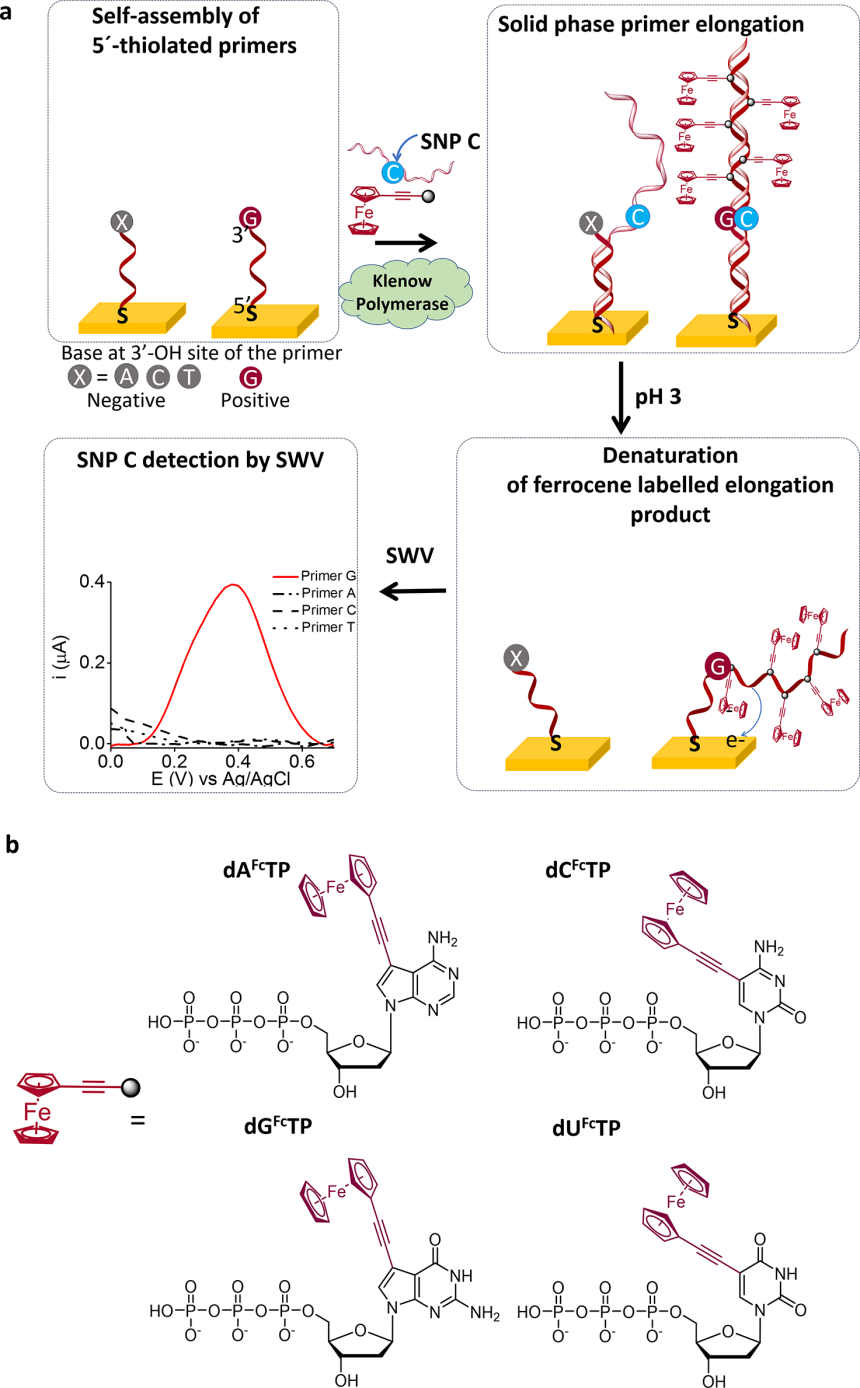
(a) Schematic representation of the solid-phase primer elongation approach with the SWV corresponding to fully complementary (terminal base = G) and nonfully complementary primers (terminal bases = A, C, or T). (b) Ferrocene-labeled dNTPs (dN^Fc^TP) used for primer elongation.

We selected a 128 mer oligonucleotide that includes an 81 nt RIF-resistance determining region (RRDR) of the beta subunit of the RNA polymerase (rpoB) gene plus primer regions as a model target. The concept is based on the use of four primers (Table S1), designed to be complementary to a 24 nt fragment of the target sequence and differing only in the terminal base at the polymorphic site. These 5′-thiolated primers are immobilized on individual gold electrodes, and following hybridization with a single-stranded DNA target, only the fully complementary primer should be elongated. The primer elongates by incorporating redox-labeled nucleotides, which can subsequently be electrochemically detected, thus facilitating identification of the SNP under interrogation (Figure 1a).

From the portfolio of our modified dNTPs bearing polyoxometalates,^54^ benzofurazane, nitrobenzene,^55,56^and other electroactive substituents, we selected ferrocenylethynyl derivatives of dNTPs, which are available with all four nucleobases (Figure 1b).^49−51,57,58^

**Figure 2:**
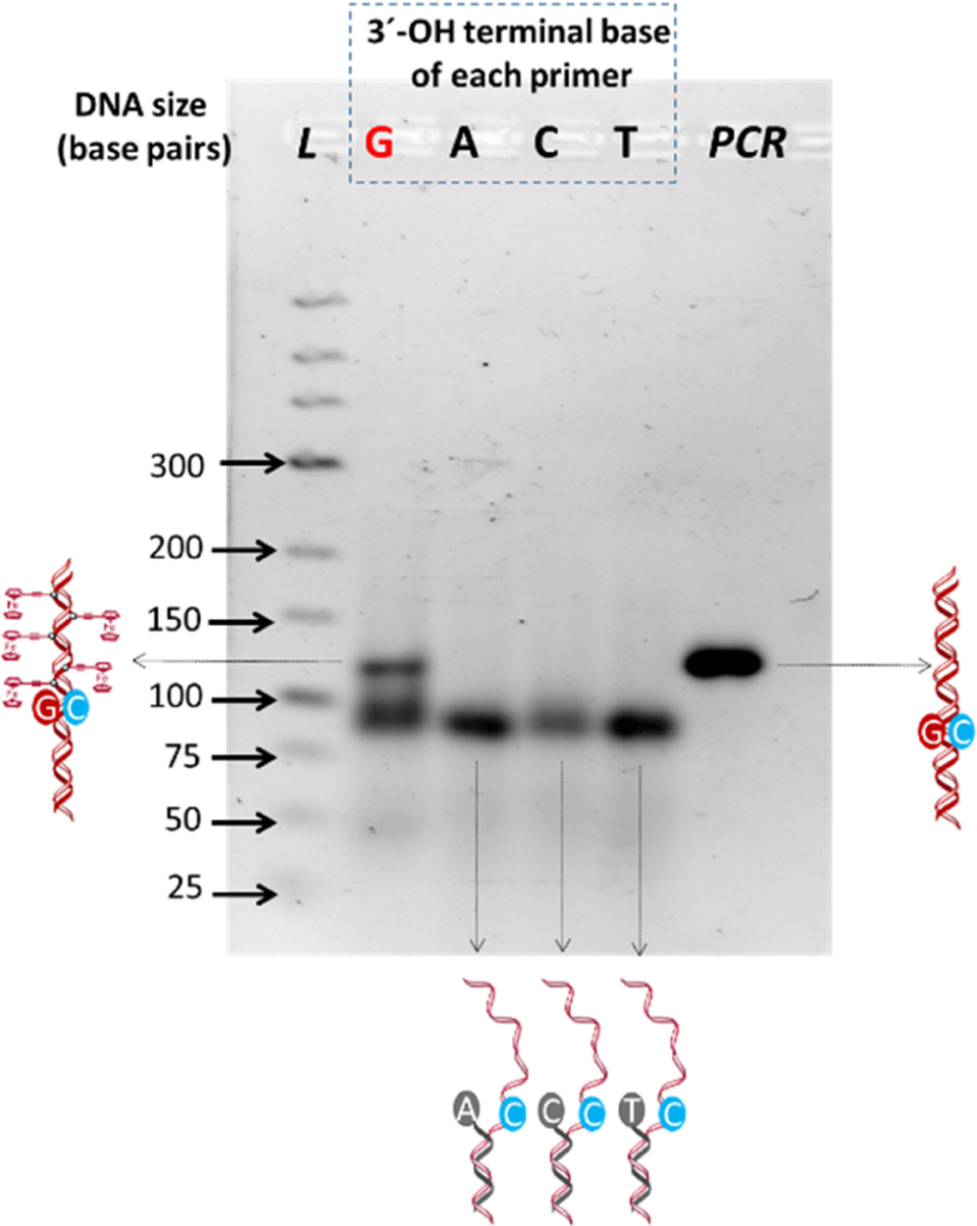
Agarose gel electrophoresis after 25 min solution-phase primer elongation for fully complementary (terminal base = G) and nonfully complementary primers (terminal base = A, C, or T). PCR: PCR product used as a positive control for elongation (prepared using natural dNTPs, as described in the Supporting Information). L: DNA ladder used to indicate the DNA size.

We initially carried out solution-phase primer elongation using 30% of all four dN^Fc^TPs (7.5% of dA^Fc^TP + 7.5% dC^Fc^TP + 7.5% dG^Fc^TP + 7.5% dU^Fc^TP of the total amount of dNTP in the reaction; 0.06 mM of each dN^Fc^TP) in a mixture with the 70% of the natural dNTPs (17.5% of dATP + 17.5% dCTP + 17.5% dGTP + 17.5% dUTP of the total amount of dNTP in the reaction; 0.140 mM of each natural dNTP) to compare isothermal vs thermocycled primer elongation. We evaluated two enzymes known to incorporate modified nucleotides: Kapa2G Robust DNA polymerase^54^ for thermocycled primer elongation and Klenow (exo-) DNA polymerase^49^ for isothermal primer elongation. The products were visualized using electrophoresis (Figures 2 and S2), and it can be clearly seen that the band corresponding to a primer elongation product is only obtained for primer G (fully complementary with the SNP C).

The tolerance of the Klenow (exo-) DNA polymerase to enzymatically incorporate different levels of the ferrocene-labeled nucleotides as substrates was evaluated using solution-phase primer elongation with each individual labeled dN^Fc^TP. Different reaction mixtures containing three native dNTPs and a fourth dNTP mixed with increasing percentages of labeled dN^Fc^TP were evaluated. The master mix contains the mixture of dNTPs (concentrations of the labeled and unlabeled dNTPs are detailed in Table S2), one primer, which is fully complementary with the target, the ssDNA target, and the Klenow (exo-) DNA polymerase, as described in Section 3 of the Supporting Information and Figure S4.

**Figure 3:**
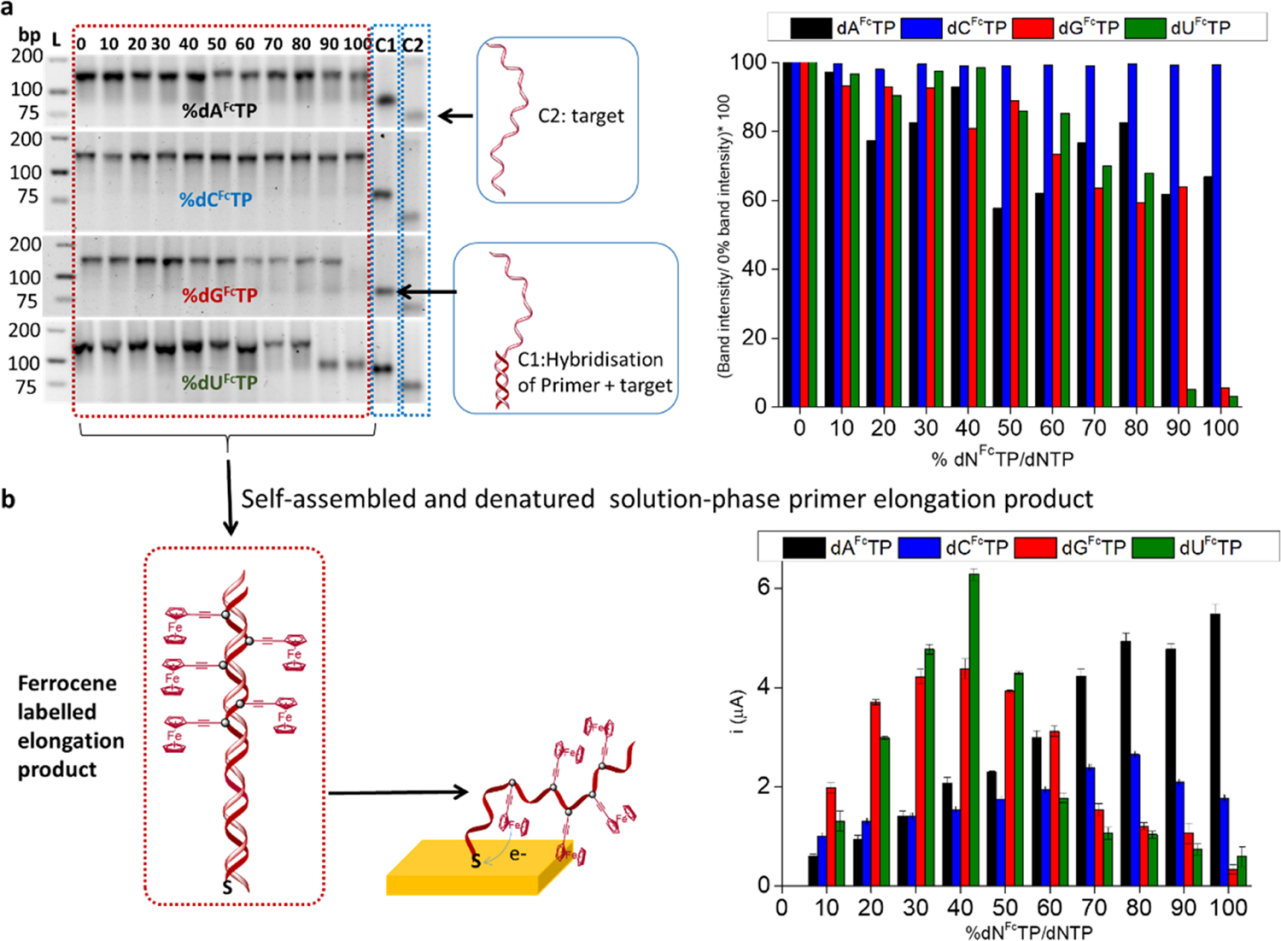
(a) (Left) 4% agarose gel electrophoresis after primer elongation using different percentages of each individual dN^Fc^TP (from 0 to 100%) and the corresponding controls (C1: hybridized primer + target in the absence of enzyme and C2: target), L: ladder. (Right) Gel electrophoresis band intensities estimated using the ImageJ program. (b) Intensities of the SWV signals of the solution-phase elongation product self-assembled on the electrode. The measurements were carried out in triplicate.

The agarose electrophoresis gels for the different percentages of each individual dN^Fc^TPs are shown in Figures 3a and S5. Two negative controls were included (C1: the target hybridized to the primer (Rv primer + target in the absence of the enzyme) to avoid confusion with the elongated products arising from their similar molecular weights and C2: the target in solution). As can clearly be observed, the yield of the reaction depends on the percentage of the modified nucleotide present in the reaction mixture. The intensity of the bands for each electrophoresis gel was plotted individually in Figure 3a (right). As observed, for dA^Fc^TP and dC^Fc^TP, the yields of the reactions appear to be unaffected, whereas, for dG^Fc^TP and dU^Fc^TP, lower yields of elongation products were obtained with the increase in the amount of ferrocene-labeled dNTP.

However, the number of ferrocene-labeled nucleotides enzymatically incorporated cannot be elucidated from the gel. The compromise between the decrease in the elongation efficiency due to the bulkier Fc and the increase in the molecular weight of the elongated Fc product due to the presence of Fc hinders the interpretation of the results. Since the presence of Fc in the DNA can be unequivocally confirmed by its oxidation wave in the SWV of the products, the solution-phase primer elongation was carried out using 5′-thiolated primers to allow their postelongation immobilization via chemisorption, as described in Section 3 of the Supporting Information and schematically shown in Figure S4. The elongation products were column purified to eliminate free dN^Fc^TPs, Klenow (exo-) DNA polymerase, and nonelongated primers, then reconstituted in the same volume of buffer and self-assembled on gold electrodes. Following chemisorption, the dsDNAs were denatured to eliminate any dN^Fc^TP intercalated into a duplex, which also provided increased flexibility of the surface-tethered elongated primer, allowing closer proximity of the ssDNA to the electrode and enhancing electron transfer. The gold electrodes were thus only modified by the elongated thiolated strand, and the electrochemical signal recorded was only provided by the ferrocene moiety of the ferrocene-labeled nucleotides enzymatically incorporated during the elongation process.

Figure S5 shows the SWV oxidation peaks for the different percentages of individual dN^Fc^TP (in mixtures with the same natural dNTP) incorporated in the elongated product, and a comparison of the peak intensities can be seen in Figure 3b. As can be observed, in the case of dA^Fc^TP, the intensity of the signal increased with the percentage of dA^Fc^TP in the mixture up to 100%, while the intensity of the signal decreased from 80% for dC^Fc^TP, 50% for dG^Fc^TP, and 40% for dU^Fc^TP.

**Figure 4:**
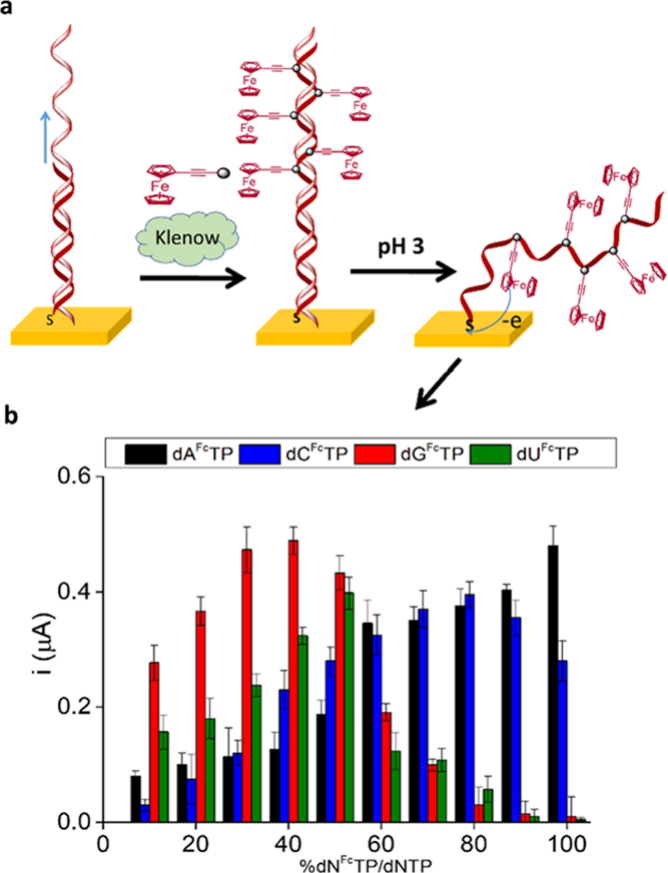
(a) Schematics of solid-phase primer elongation and denaturation of the solid-phase elongation product at acidic pH. (b) Intensities of the SWV signals of solid-phase elongation products. The measurements were carried out in triplicate.

A similar study was carried out using solid-phase primer elongation with immobilized primers again varying the percentages of each of the dN^Fc^TPs (Figure S6). A bipodal PEGylated alkanethiol^48^ was coimmobilized with primers, acting as a backfiller to space the primers on the surface,^59,60^ allowing the hybridization of target and the accessibility of the Klenow (exo-) DNA polymerase. As can be observed in Figure 4, dA^Fc^TP was incorporated even at 100%, with dC^Fc^TP being effectively incorporated up to 80%, while no signal was observed for either dG^Fc^TP or dU^Fc^TP at levels higher than 50%, correlated to the results obtained using solution-phase primer elongation. This may be attributable to a better substrate specificity of the Klenow (exo-) DNA polymerase for dA^Fc^TP as compared to dG^Fc^TP and dU^Fc^TP, as well as the possible steric hindrance between consecutive ferrocene-labeled bases at homopolymeric regions of the amplicon, which could inhibit the elongation at higher percentages of dN^Fc^TP, as was previously observed for other bulky electroactive labels.^54^

**Figure 5:**
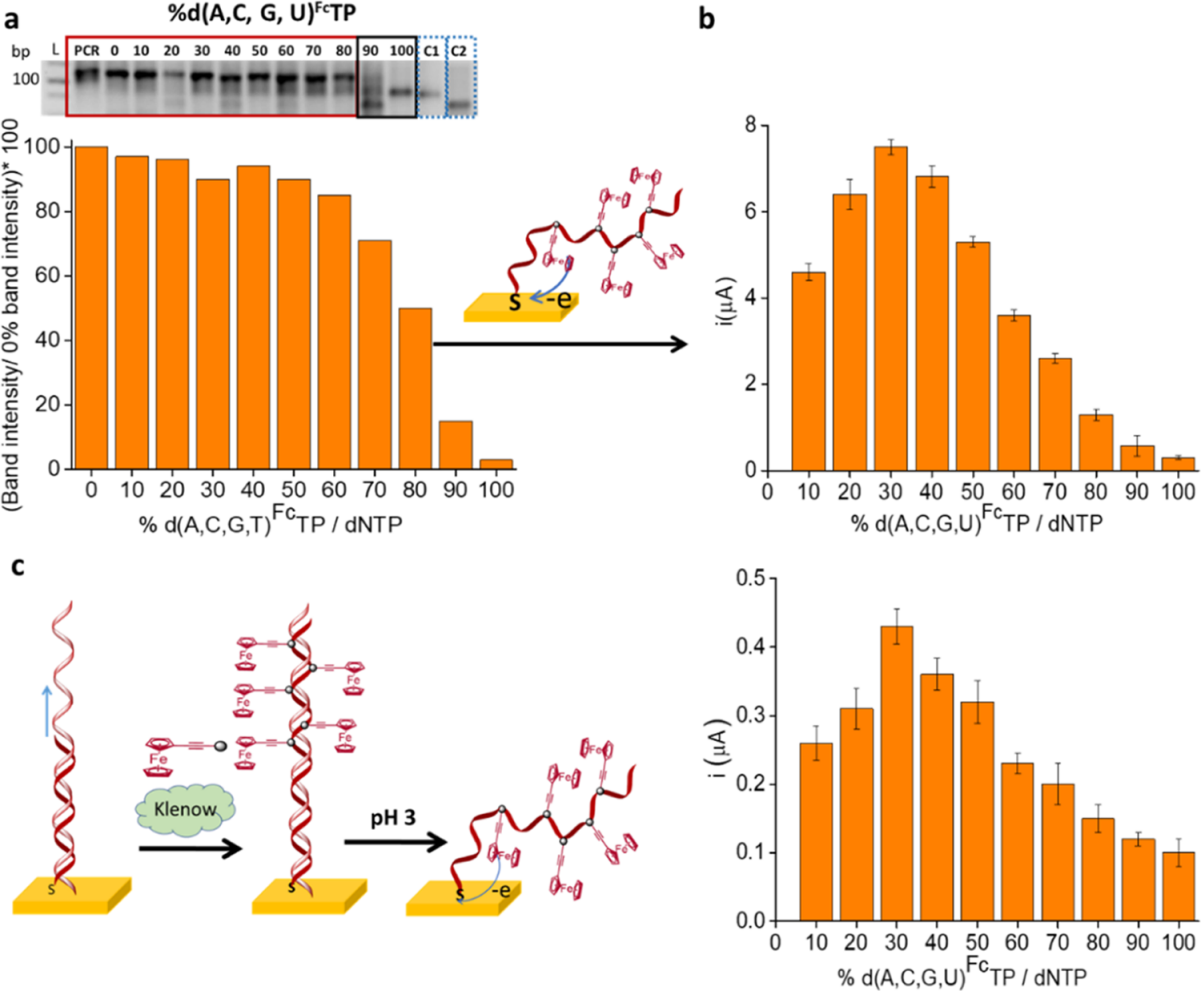
(a) 4% agarose gel electrophoresis after primer elongation using different percentages of the mixture of the four dN^Fc^TP (from 0 to 100%) and the corresponding controls (C1: hybridized primer + target and C2: target), L: ladder. The intensities of the bands were calculated using the ImageJ program. (b) Intensities of the SWV signals of the solution-phase elongation product self-assembled on the electrode. (c) Intensities of the SWV signals of solid-phase elongation products. The measurements were carried out in triplicate.

Due to the target dependence of the signal, and to generalize the approach for use with diverse sequences, we explored the option to use mixtures of all four labeled dNTPs containing each of the labeled bases at identical percentages, while maintaining the total nucleotide concentration. As described previously, in the first instance, solution-phase primer elongation using thiolated primers was carried out and the elongation products were analyzed using electrophoresis, and following purification, chemisorption onto gold electrodes, and duplex denaturation, SWV was carried out. Different percentages of the mixture of all four labeled dNTPs with natural dNTPs, (dA^Fc^TP + dC^Fc^TP + dG^Fc^TP + dU^Fc^TP)/(dATP + dCTP + dGTP + dTTP), were added to the master mix with the target, enzyme, and the primer fully complementary with the target. As can be seen in the gel image in Figure 5a, the elongation reaction functioned up to at least 70% of dN^Fc^TPs/dNTPs (17.5% of dA^Fc^TP + 17.5% dC^Fc^TP + 17.5% dG^Fc^TP + 17.5% dU^Fc^TP in the reaction; 0.140 mM of each dN^Fc^TP), and increasing the percentage of dN^Fc^TPs resulted in a decreasing size of the elongation product, with an undefined band observed at 80% (20% of dA^Fc^TP + 20% dC^Fc^TP + 20% dG^Fc^TP + 20% dU^Fc^TP in the reaction; 0.160 mM of each dN^Fc^TP), poor elongation obtained for 90% (22.5% of dA^Fc^TP + 22.5% dC^Fc^TP + 22.5% dG^Fc^TP + 22.5% dU^Fc^TP in the reaction; 0.180 mM of each dN^Fc^TP), and 100% (25% of dA^Fc^TP + 25% dC^Fc^TP + 25% dG^Fc^TP + 25% dU^Fc^TP in the reaction; 0.2 mM of each dN^Fc^TP). As observed in Figure 5b, the highest electrochemical signal was observed with 30% dN^Fc^TPs/dNTPs (7.5% of dA^Fc^TP + 7.5% dC^Fc^TP + 7.5% dG^Fc^TP + 7.5% dU^Fc^TP in the reaction; 0.06 mM of each the dN^Fc^TP). The experiment was repeated for solid-phase primer elongation with varying percentages of dN^Fc^TPs/dNTPs, and again, 30% of the labeled dNTPs resulted in the highest electrochemical signal. The surface confinement of the ferrocene- labeled nucleotides was confirmed by the linearity of the plot of the intensity of the oxidation and reduction peaks with the scan rate (Figure S8). The use of the 30% mixture of all four labeled dN^Fc^TPs, containing an equimolar ratio of each dN^Fc^TPs, not only has the advantage of giving the highest signal but is additionally more universally applicable to any sequence, avoiding any potential problems in sequences heavy in the homopolymeric content.

Using 30% of all four dN^Fc^TPs/dNTPs, the primer elongation time was optimized to maximize the discrimination between specific and nonspecific primer elongation. Each of the 5′-thiolated reverse primers was immobilized on individual gold electrodes of an array. Following 20 min of hybridization of the target, primer elongation was allowed to proceed for 2, 5, 10, and 15 min, followed by denaturation and SWV measurement in Tris buffer. Overall, 2–5 min were sufficient to obtain specific signals, whereas longer elongation times resulted in the generation of nonspecific signals (Figure S7a). As previously observed,^49^ the low working temperature of Klenow (exo-) DNA polymerase may decrease the precision of the enzyme, leading to the increase in its tolerance to incorporate noncomplementary bases with extended reaction time, which is enhanced by the high degree of complementarity of the primers with the target DNA.

For the reliable detection of SNPs, reaction specificity is critical, and the optimum reaction time based on the difference between the positive signal (primer G) and the average of the three negative controls (non-full complementary primers A, C, and T) was found to be 5 min (Figure S7b). Higher and lower hybridization times were also evaluated, but no improvement of the signal was observed (Figure S7c,d). Therefore, 20 min hybridization time and 5 min primer elongation were considered as optimum for SNP detection, allowing a significant discrimination between positive and negative signals, as shown in the inset of Figure 1.

SNP detection was carried out using a range of concentrations of target DNA with all of the best combinations of labeled and native dNTPs (100% dA^Fc^TP, 80% dC^Fc^TP, 40% dG^Fc^TP, 50% dU^Fc^TP, and 30% × (d(A,C,G,U)^Fc^TP)). All combinations allowed reliable detection of SNPs at low picomolar levels of target DNA, with the mixture of 30% × (d(A,C,G,U)^Fc^TP = 7.5% of each dN^Fc^TP and 70% of unlabeled dNTPs), allowing detection and identification of the SNP at just 3 pM (2.7 × 10^6^ DNA copies) of the target DNA containing the SNP to be interrogated (Figure S9 and Table S3). Although lowering the detection limit is a goal, this method has been successfully applied without further optimizations to the detection of SNPs in samples of biological origin.

A total of 14 samples of genomic DNA extracted from *Mycobacterium tuberculosis* strains were analyzed using this approach. First, ssDNA was produced following a procedure described in Section 4.3 of the Supporting Information and Figure S10. The ssDNA was used for hybridization, solid-phase primer elongation, and further electrochemical detection (Figure S11). The unequivocal discrimination between positive and negative signals supported the robustness of the method. The base detected at the SNP site (positive primer A, SNP T) correlated with the results obtained by sequencing.

## Conclusions

The electrochemical detection of solid-phase primer elongation from gold electrode surfaces using ferrocene-labeled oligonucleotides has been exploited for the detection of single-nucleotide mutations.

In this first proof-of-concept, we have focused on the detection of a single SNP associated with rifampicin resistance. Individual electrodes of an array were functionalized with thiolated primers identical with the exception of their 3′ terminal base. Following a 20 min hybridization with the target DNA containing the SNP site to be interrogated, a 5 min Klenow (exo-) DNA polymerase-mediated solid-phase primer elongation with ferrocene-labeled oligonucleotides resulted in an unequivocal identification of the SNP, even at low picomolar concentrations. The approach was validated by the successful SNP discrimination in 14 samples containing DNA extracted from *Mycobacterium tuberculosis* strains.

While the current study concentrated on a single SNP, the platform is inherently compatible with multiplexed detection, where electrodes of an array can be functionalized with primers for different SNPs, with the number of SNPs to be analyzed dictating the number of the electrodes in the array to be used.

To date, the majority of multiplexed SNP microarray platforms are mainly based on fluorescence detection with CCDs inherently requiring cooling and complex optics. Most platforms also require sample processing/treatment, e.g., extraction of DNA, amplification of DNA, and generation of single-stranded DNA. The use of electrochemical detection as an alternative to fluorescence detection, which is compatible with handheld potentiostats such as those used in glucometers, can thus facilitate portability and cost-effectiveness, moving the multiplexed detection of SNPs closer to the point-of-need. Although many of the developed microarray platforms are aimed at whole-genome analysis, this generic electrochemical platform can find applications where a subset of application-specific SNPs need to be analyzed in a rapid, cost-effective, and facile manner. Incorporating this platform within a simple microfluidic system can find widespread applications in many diverse areas, including advanced forensics, patient stratification, screening for disease predisposition, as well as in crop genetics and the identification of antibiotic resistance.

## Supporting Information Available

Experimental details, raw data, and optimization experiments (PDF)
