# Supplementary material for: Electrochemical Detection of Single-Nucleotide Polymorphism Associated with Rifampicin Resistance in Mycobacterium tuberculosis Using Solid-Phase Primer Elongation with Ferrocene-Linked Redox-Labeled Nucleotides
Source: ACS Sens. 2021 Nov 19;6(12):4398–407. doi: 10.1021/acssensors.1c01710 (PMC8715531; doi:10.1021/acssensors.1c01710)
Supplement: Supplementary file 1 — se1c01710_si_001.pdf [file se1c01710_si_001.pdf]

Supporting Information

Electrochemical detection of single nucleotide polymorphism associated with rifampicin resistance in *Mycobacterium tuberculosis* using solid-phase primer elongation with ferrocene-linked redox-labelled nucleotides

Mayreli Ortiz,[a] Miriam Jauset-Rubio,[a] Vasso Skouridou,[a] Diana Machado,[b] Miguel Viveiros,[b] Taane G. Clark,[b,c] Anna Simonova,[d,e] David Kodr,[d] Michal Hocek,[d,e]* and Ciara K. O'Sullivan[a,f]*

[a] Departament d'Enginyeria Química, Universitat Rovira i Virgili, Avinguda Països Catalans 26, 43007 Tarragona, Spain.

[b] Global Health and Tropical Medicine, GHTM, Instituto de Higiene e Medicina Tropical, IHMT, Universidade Nova de Lisboa, Rua da Junqueira, 100,
1349-008 Lisbon, Portugal

[c] Faculty of Infectious and Tropical Diseases, London School of Hygiene & Tropical Medicine, WC1E 7HT London, UK

[d] Institute of Organic Chemistry and Biochemistry, Czech Academy of Sciences, Flemingovo nam.2, 16610 Prague 6, Czech Republic.

[e] Department of Organic Chemistry, Faculty of Science, Charles University, Hlavova 8, 12843 Prague 2, Czech Republic

[f] Institució Catalana de Recerca i Estudis Avançats, Passeig Lluis Companys 23, 08010 Barcelona, Spain

*Corresponding author at: Institute of Organic Chemistry and Biochemistry, Czech Academy of Sciences, Flemingovo nam.2, 16610 Prague 6, Czech Republic. E-mail address: hocek@uochb.cas.cz (M. Hocek)

**Corresponding author at: Departament d'Enginyeria Química, Universitat Rovira i Virgili, Avinguda Països Catalans 26, 43007 Tarragona, Spain. E-mail address: ciara.osullivan@urv.cat (C.K. O´Sullivan).

Table of content:

Additional experimental details. Figures and Tables 3

1. Electrochemical cleaning of electrodes. Experimental details. 4

Figure S1. (a) Gold electrode array used for the experiments. The array is composed by 16 individual 1 mm^2 square gold working electrodes (150 nm in thick) with a common gold counter electrode and silver reference electrode. (b) Overlapping of cyclic voltammograms recorded in sulfuric acid of the 16 working electrodes. (c) Details of the gold reduction peaks to illustrate the reproducibility of the working electrode areas. 4

2. PCR and Thermo-cycled primer elongation reaction using Kapa2G Robust DNA polymerase. Experimental details. 4

2.1. PCR amplification 4

2.2. Thermo-cycled primer elongation reaction 5

Figure S2. 4% agarose gel electrophoresis after 20 cycles of thermo-cycled primer elongation reaction using Kapa2G Robust DNA polymerase and four 5'-thiolated-primers with different base at 3'-OH (A, C, G or T). L: DNA ladder. 5

Figure S3. (a) Ferrocene-2'-deoxyribonucleoside triphosphates ($dN^{Fc}TP$). (b) Square wave voltammograms (SWV) of individual $dN^{Fc}TP$ recorded in 10 mM Tris + 0.1 M KCl (pH 7.4).............6

3. Optimisation of the percentage of $dN^{Fc}TP$ using liquid-phase primer elongation 6

Table S2. Concentrations of $dA^{Fc}TPs$ and unlabelled dNTPs used in primer elongation reactions to evaluate effect of increasing percentages of $dA^{Fc}TPs$. 6

Figure S4. Schematic representation of the flow through for the study of primer extension using Klenow DNA polymerase and $dN^{Fc}TP$. 8

Figure S5. Left: Agarose gel electrophoresis for primer elongation products using different percentages of each $dN^{Fc}TP$ in the reaction mixture. (a) $dA^{Fc}TP$, (b) $dC^{Fc}TP$, (c) $dG^{Fc}TP$, (d) $dU^{Fc}TP$, (e) $d(A,C,G,U)^{Fc}TP$. The reaction using only natural dNTP was considered as positive control providing maximum elongation yield, while target (T) and target and primer just hybridised (T+P) were included in the gel to help with the identification of the elongation product. Right: SWVs recorded for the immobilised Fc-primer elongation products on electrode surfaces...9

4. Optimisation of solid phase primer elongation reaction...10

4.1 Optimisation of the percentage of the labelled bases in the master mix. 10

Figure S6. SWVs recorded after solid phase primer elongation reaction for each base (a) $dA^{Fc}TP$, (b) $dC^{Fc}TP$, (c) $dG^{Fc}TP$, (d) $dU^{Fc}TP$ and (e) $d(A, C, G, U)^{Fc}TP$ at different percentage in the reaction mixture (from 10 to 100%). 10

4.2 Optimisation of the solid phase primer elongation time and hybridisation time. Experimental details. 10

Figure S7. (a) Optimisation of the time of primer elongation reaction after 20 min of hybridisation, based on the SWV signal from the positive signal (Primer G) and three negative controls (non-full complementary primers (A, C and T)). (b) Difference in current intensities between the positive signal (primer G) and the average of the three negative controls (non-full complementary primers A, C and T) vs. reaction time of the primer elongation reaction. (c) Optimisation of the hybridisation time of primer elongation reaction based on the SWV signal from the positive signal (Primer G) and three negative

controls (non-fully complementary primers (A, C and T)) followed by 5 min primer elongation. (d) Difference in current intensities between the positive signal (primer G) and the average of the three negative controls (non-full complementary primers A, C and T) vs. hybridisation time.......................11

Figure S8. (a) Cyclic voltammograms at different scan rates of surface confined Fc-containing elongation product in 10 mM Tris + 0.1 M KCl. (b) Peak currents vs scan rate plot. 12

Figure S9. Differential signal over range of DNA concentrations. Signals measured using a range of concentrations of target DNA showing the differentiation between positive signal i.e. primer extension from the immobilised primer with the terminal base complementary to the SNP in the target and negative signals i.e. negligible primer extension from the immobilised primers with terminal bases not complementary to the SNP in the target. 12

Table S3. Parameters calculated from calibration curves using the best combinations of labeled and natural dNTPs using Sigmoidal four-parameter logistic (4PL) fitting. The LOD indicates the minimum concentration for differential identification of the SNP under interrogation. 13

4.3 Validation of the assay using real samples. 13

4.3.1 PCR amplification and ssDNA generation. 13

Figure S10. Agarose gel electrophoresis (2.6 % m/v) after different steps of ssDNA generation from DNA extracted from real samples of *Mycobacterium tuberculosis*: a) PCR using forward primer and phosphorylated reverse primer; b) Asymmetric PCR; c) Lambda exonuclease digestion and d) The corresponding NTC of a), b) and c).(L: Ladder, bp: base pairs). 14

4.3.2 Electrochemical detection of samples 14

Figure S11. SWVs recorded after solid phase primer elongation reaction using d(A, C, G, U)^{Fc}TP (all bases at 30%) in the reaction mixture of 14 samples containing DNA extracted from *Mycobacterium tuberculosis*...15

Additional experimental details. Figures and Tables

Table S1. The HPLC purified oligonucleotides used for the study.

Sequences used for general approach	
Target *M. tuberculosis* mut rpoB (128 nt)	5'cgatcaaggagttcttcggcaccagccagctgagccaattcatggaccagaacaacc cgctgtcggggttgacccacaagcgccgactgtcggcgc***C***ggggcccggcggtctgt cacgtgagcgtgcc-3'
Primer G → 3'-***G*** (fully complementary primer)	5'-HS-ttt-cacgtgacagaccgccgggcccc***G***-3'
Primer A → 3'-***A***	5'-HS-ttt-cacgtgacagaccgccgggcccc***A***-3'
Primer C → 3'-***C***	5'-HS-ttt-cacgtgacagaccgccgggcccc***C***-3'
Primer T → 3'-***T***	5'-HS-ttt-cacgtgacagaccgccgggcccc***T***-3'
Forward Primer (for PCR amplification)	5'-cgatcaaggagttcttcggc-3'
Phosphorylated Primer (for PCR amplification)	5'-cacgtgacagaccgccgggcccc-3'

1. Electrochemical cleaning of electrodes. Experimental details.

Firstly, the electrode arrays were gently rinsed with acetone and sonicated in isopropanol, acetone and water for 5 min each to remove a protective lacquer and dried with nitrogen. Then, the electrode arrays were electrochemically cleaned using cyclic voltammetry, firstly applying a reductive desorption in basic media (KOH, 0.1 M) from 0 to -1.2 V vs Ag, with a scan rate of 100 mV/s for 10 scans. After washing with MilliQ water, the electrodes were electrochemically cleaned again by cycling 10 times the potential from 0 to 1.8 V vs Ag in acid media (H_2SO_4, 0.1 M), with a scan rate of 100 mV/s. Finally, the arrays were washed with water and dried with nitrogen for further functionalisation.

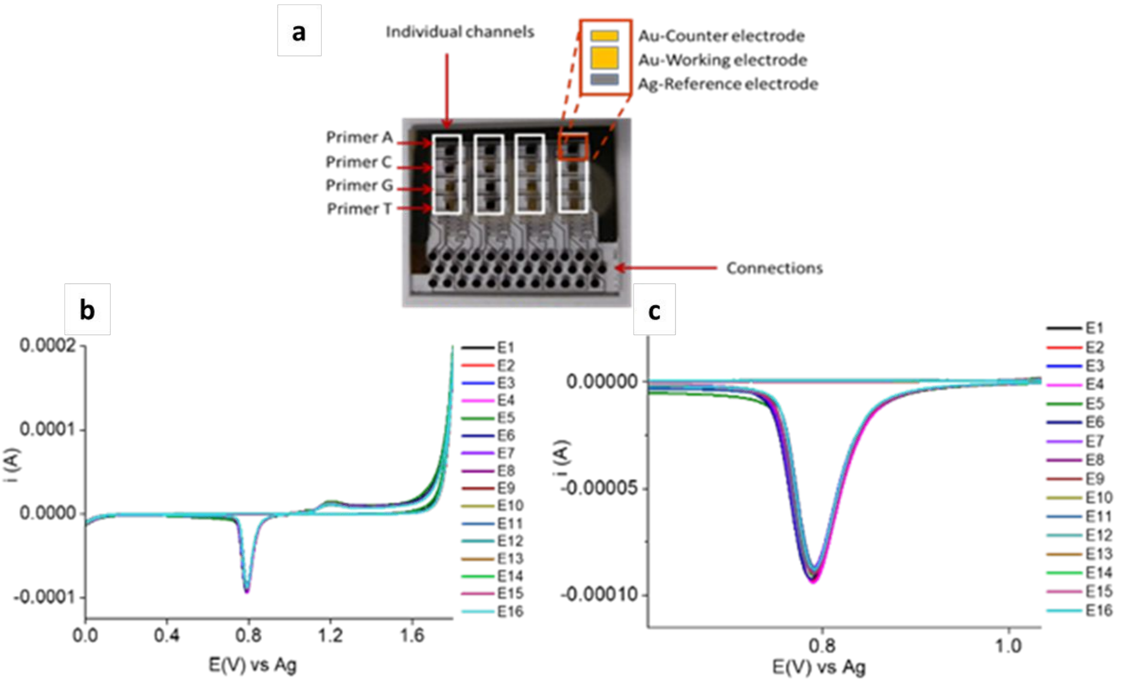

Figure S1. (a) Gold electrode array used for the experiments. The array is composed by 16 individual 1 mm^2 square gold working electrodes (150 nm in thick) with a common gold counter electrode and silver reference electrode. (b) Overlapping of cyclic voltammograms recorded in sulfuric acid of the 16 working electrodes. (c) Details of the gold reduction peaks to illustrate the reproducibility of the working electrode areas.

2. PCR and Thermo-cycled primer elongation reaction using Kapa2G Robust DNA polymerase. Experimental details.

2.1. PCR amplification

PCR amplification was carried out to be used as positive control for primer elongation. Fifty microlitres of master mix containing 0.2 µM of the fully complementary reverse primer (G) and 0.2 µM of forward primer, 1 X KAPA2G Robust PCR buffer, 1 X KAPA2G Robust enhancer, 200 µM of natural dNTP

and 1 unit of the KAPA2G Robust DNA polymerase was prepared with 100 pM target sequence. PCR reaction was carried out in an iCycler Thermal Cycler (Biorad) with the following conditions: 2 min at 95ºC, followed by 20 cycles composed by three steps: 1) denaturation during 30 s at 95ºC, 2) annealing during 30 s at 58ºC and elongation during 30 s at 72ºC and a final elongation step for 5 min at 72ºC. At the end of the program, the tubes were stored at 4ºC.

2.2. Thermo-cycled primer elongation reaction

Fifty microlitres of master mix containing 0.4 µM of the corresponding reverse primer (A, C, G or T), 1 X KAPA2G Robust PCR buffer, 1 X KAPA2G Robust enhancer, 200 µM of dNTP (containing 30% molar $dN^{Fc}TP$ (7.5% of $dA^{Fc}TP$ + 7.5% $dC^{Fc}TP$ + 7.5% $dG^{Fc}TP$ + 7.5% $dU^{Fc}TP$ of total amount of dNTP in the reaction) in a mixture with the 70% of the natural dNTPs (17.5% of dATP + 17.5% dCTP + 17.5% dGTP + 17.5% dTTP of total amount of dNTP in the reaction)) and 1 unit of the KAPA2G Robust DNA polymerase was prepared with 100 pM target sequence. Primer elongation was carried out in an iCycler Thermal Cycler (Biorad) with the following conditions: 2 min at 95ºC, followed by 20 cycles composed by three steps: 1) denaturation during 30 s at 95ºC, 2) annealing during 30 s at 58ºC and elongation during 30 s at 72ºC and a final elongation step for 5 min at 72ºC. At the end of the program, the tubes were stored at 4ºC.

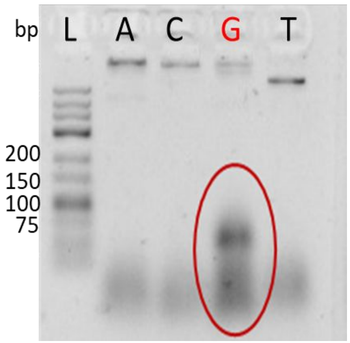

Figure S2. 4% agarose gel electrophoresis after 20 cycles of thermo-cycled primer elongation reaction using Kapa2G Robust DNA polymerase and four 5'-thiolated-primers with different base at 3'-OH (A, C, G or T). L: DNA ladder.

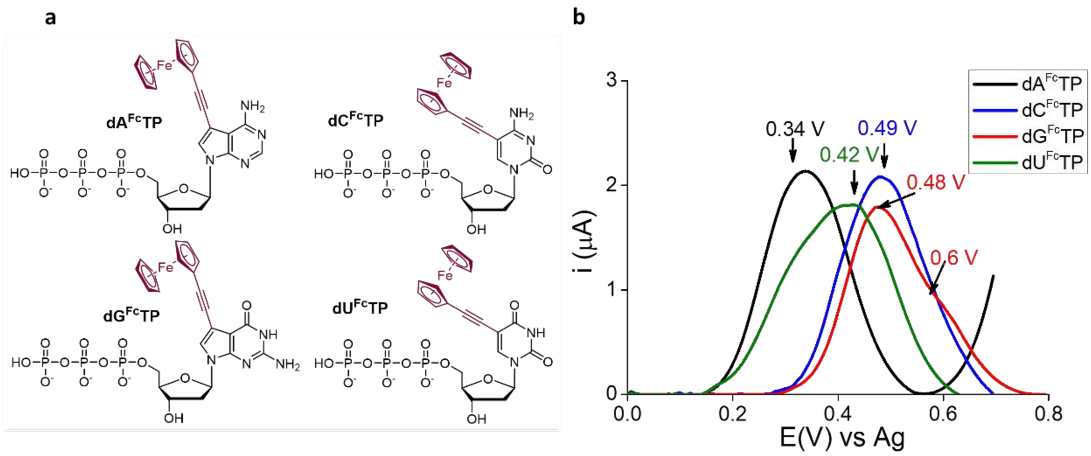

Figure S3. (a) Ferrocene-2'-deoxyribonucleoside triphosphates (dN^{Fc}TP). (b) Square wave voltammograms (SWV) of individual dN^{Fc}TP recorded in 10 mM Tris + 0.1 M KCl (pH 7.4).

3. Optimisation of the percentage of dN^{Fc}TP using liquid-phase primer elongation

The primer elongation reaction was carried out in solution using increased molar percentages of each dN^{Fc}TP (0, 10, 20, 30, 40, 50, 60, 70, 80, 90 and 100%) in the mixture of 200 µM of each dNTP also containing 0.5 µM of primer G, 1X buffer of Klenow (exo-) DNA polymerase, Klenow fragment (exo-) DNA polymerase and 0.5 µM of ssDNA template. Using the example of the dA^{Fc}TPs, Table S2 shows the concentrations of each of the dA^{Fc}TP and the unlabelled dNTPs used in each primer elongation reaction. The same can be extrapolated for each of dT^{Fc}TP, dG^{Fc}TP and dC^{Fc}TP.

Table S2. Concentrations of dA^{Fc}TPs and unlabelled dNTPs used in primer elongation reactions to evaluate effect of increasing percentages of dA^{Fc}TPs.

% dA^{Fc}TP	dA^{Fc}TP (µM)	dATP (µM)	dTTP (µM)	dCTP (µM)	dGTP (µM)
0	0	200	200	200	200
10	20	180	200	200	200
20	40	160	200	200	200
30	60	140	200	200	200
40	80	120	200	200	200
50	100	100	200	200	200
60	120	80	200	200	200
70	140	60	200	200	200
80	160	40	200	200	200
90	180	20	200	200	200
100	200	0	200	200	200

The reactions were carried out at 37°C for 25 min followed by 10 min at 60°C for enzyme deactivation. Alternatively, 10 individual solutions containing increased molar percentages of the four bases ((x% of dA^{Fc}TP + x% of dC^{Fc}TP + x% of dG^{Fc}TP + x% of dU^{Fc}TP) and (100-4x)% of each natural dNTP; with

x= 2.5, 5, 7.5, 10, 12.5, 15, 17.5, 20, 22.5, 25) were also prepared and the same procedure described above was followed (Figure S4 (II)).

Finally, 4% w/v agarose gel electrophoresis was used for detection (Schematic representation in Figure S4 (II) and gel electrophoresis Figure S5 and Figures 3a and 5a in the manuscript). The efficiency of the reactions was evaluated by comparing the intensity of the bands after gel electrophoresis which was calculated using the Gel Analysis option of the ImageJ program. After gel electrophoresis and imaging, the bands corresponding to the elongation products were selected using the rectangular selection tool as a horizontal lane and then plotted to generate a profile plot. A baseline was drawn to define the peaks in the generated plot corresponding to each band in the gel and the intensity of each peak was calculated using the wand tool. The values obtained for each peak were finally plotted against the different percentages of each $dN^{Fc}TP$ in the reaction mixture using the Origin 6.0 software.

The samples were then purified using DNA Clean & Concentrator to eliminate remaining primers, possible free-ssDNA target, Klenow (exo-) DNA polymerase and nucleotides (Figure S4 (III). All samples were recovered in the same volume (6 µL) of DNAse free water and diluted twice in 2 M KH_2PO_4 to reach a final 1 M KH_2PO_4 required for subsequent immobilisation on the electrode surface (Figure S4 (IV)). Following 3 h of immobilisation in a humidity chamber, the electrodes were generously washed with water and then incubated for 10 min with 0.1 M glycine (pH 3) to denature the dsDNA (Figure S4 (V)). After washing with glycine and water, any $dN^{Fc}TP$ intercalated in the duplex was washed away. (Figure S4 (V)). The corresponding SWVs for each $dN^{Fc}TP$ after purification and immobilisation on electrode surface are shown in Figure S5.

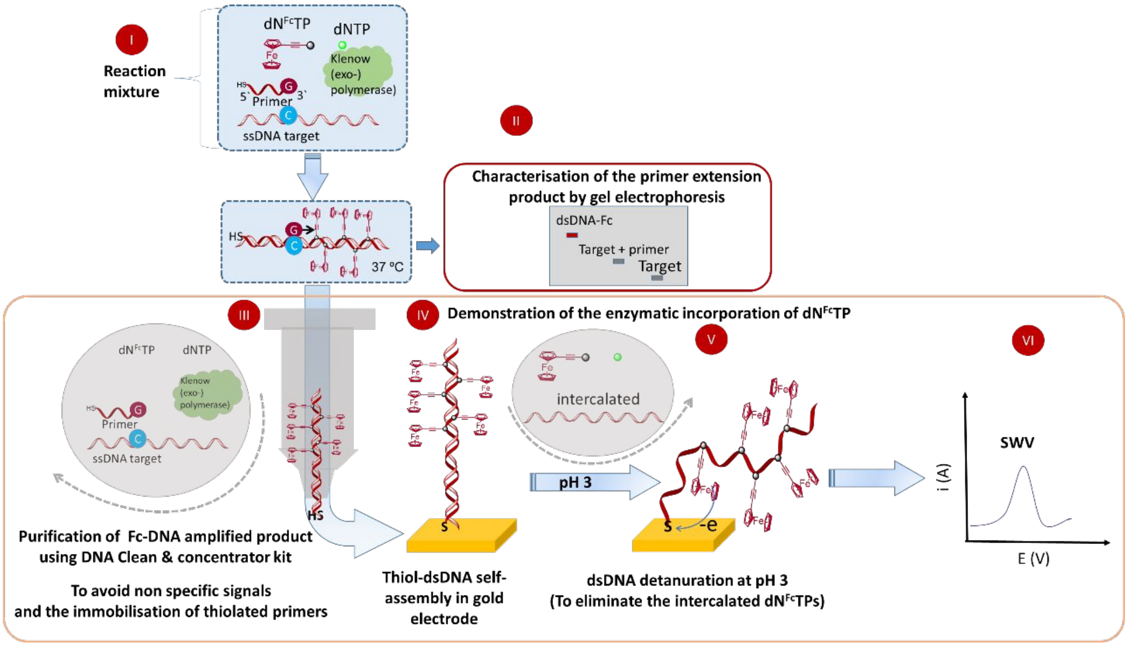

Figure S4. Schematic representation of the flow through for the study of primer extension using Klenow (exo-) DNA polymerase and dN^{Fc}TP.

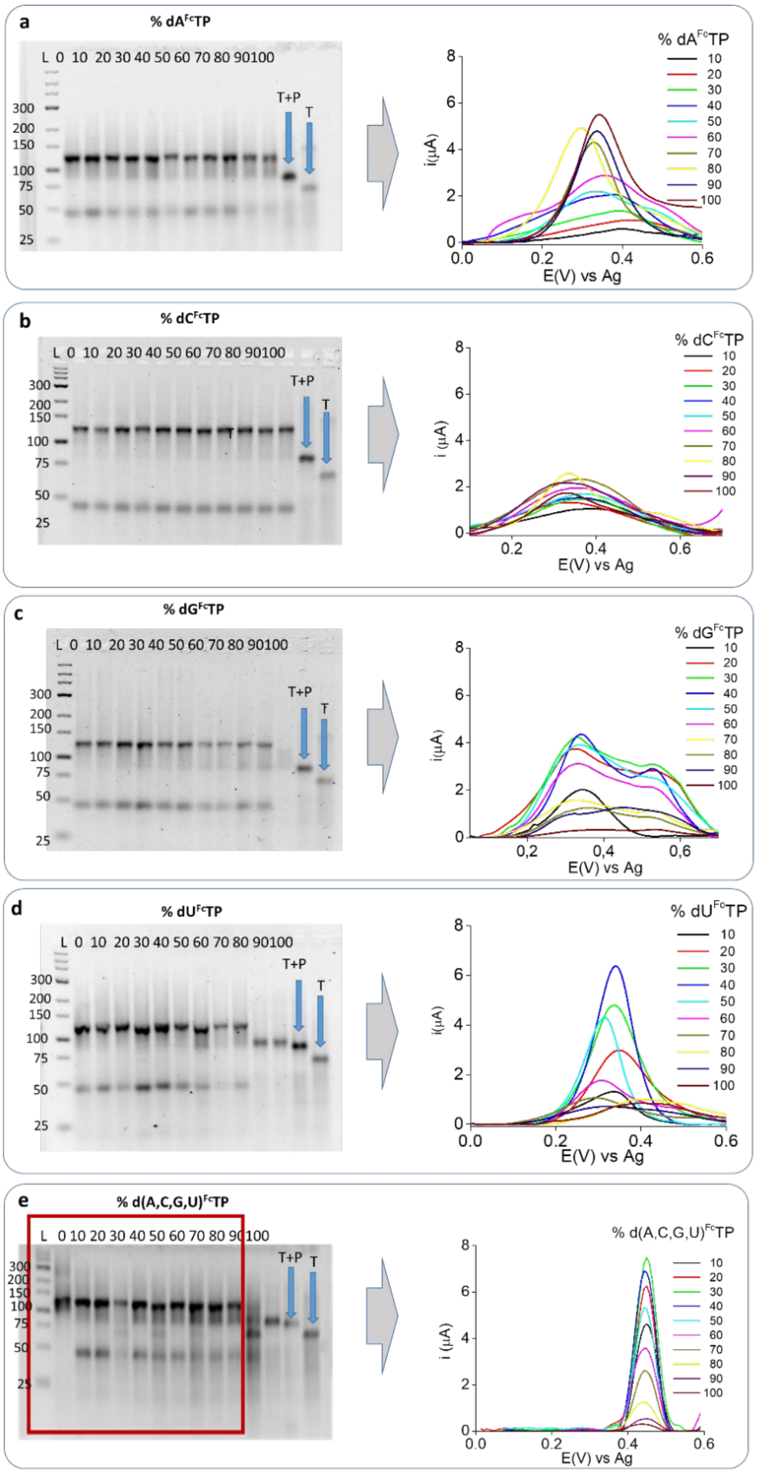

Figure S5. Left: Agarose gel electrophoresis for primer elongation products using different percentages of each $dN^{Fc}TP$ in the reaction mixture. (a) $dA^{Fc}TP$, (b) $dC^{Fc}TP$, (c) $dG^{Fc}TP$, (d) $dU^{Fc}TP$, (e) $d(A,C,G,U)^{Fc}TP$. The reaction using only natural dNTP was considered as positive control providing maximum elongation yield, while target (T) and target and primer just hybridised (T+P) were included in the gel to help with the identification of the elongation product. Right: SWVs recorded for the immobilised Fc-primer elongation products on electrode surfaces.

4. Optimisation of solid phase primer elongation reaction.

4.1 Optimisation of the percentage of the labelled bases in the master mix.

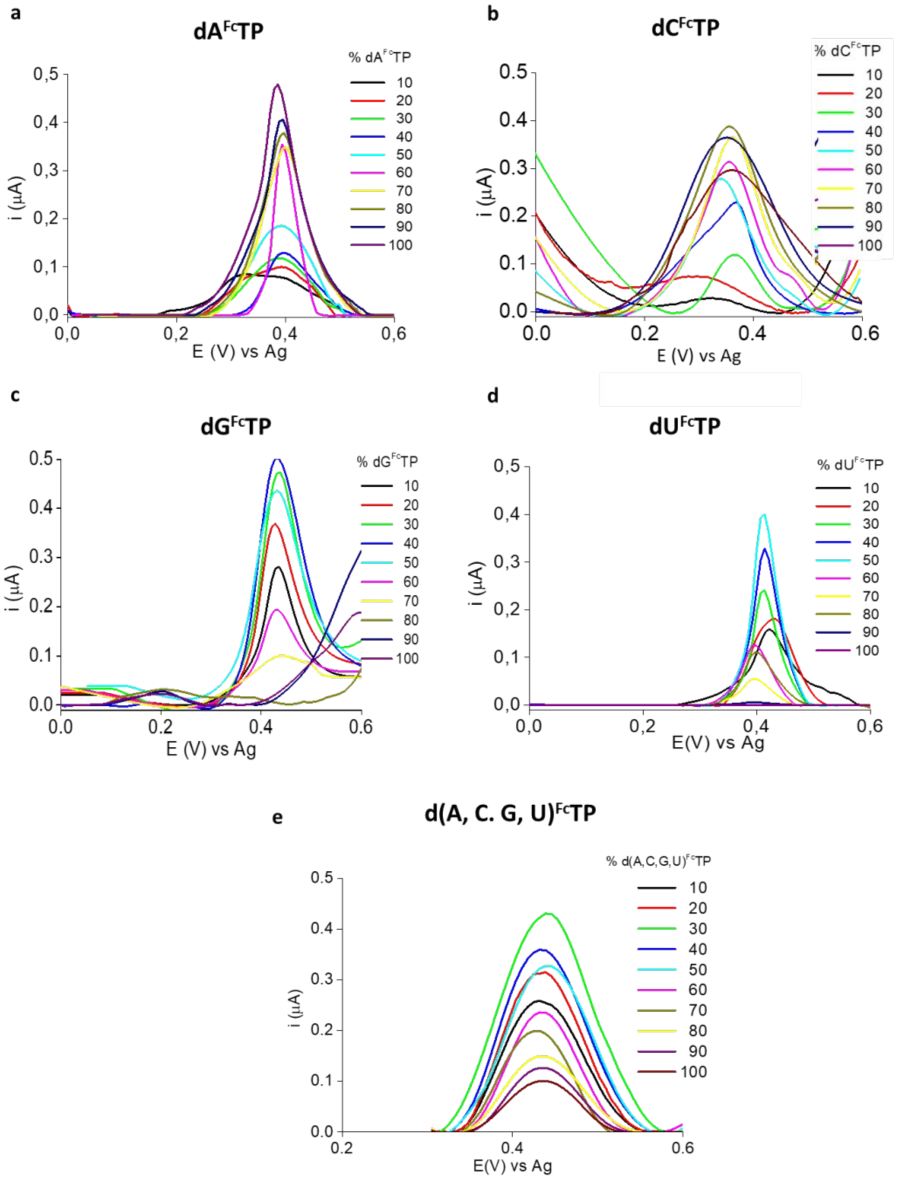

Figure S6. SWVs recorded after solid phase primer elongation reaction for each base (a) dA^{Fc}TP, (b) dC^{Fc}TP, (c) dG^{Fc}TP, (d) dU^{Fc}TP and (e) d(A, C, G, U)^{Fc}TP at different percentage in the reaction mixture (from 10 to 100%).

4.2 Optimisation of the solid phase primer elongation time and hybridisation time. Experimental details.

A microfluidic cell containing four channels was created per electrode array to perform individual reactions at different times. Fifteen microlitres of ssDNA in 10 mM Tris buffer + 500 mM NaCl, pH 7.4 were added and left to hybridise for 20 min at 37°C under shaking in a humidity chamber. Then, the

electrodes were sequentially washed with the same buffer and dried with nitrogen. 15 µL of the reaction mixture (200 µM of total dNTPs (containing 30 % $dN^{Fc}TPs$ and natural dNTPs), 1X Klenow reaction buffer and Klenow (exo-) fragment DNA polymerase) was added to each channel and left to react for different times (2, 5, 10 and 15 min) at 37°C under shaking. The electrodes were washed twice again with the buffer, the channel filled, and the electrochemical measurements carried out using SWV (Figure S7 a and b).

Once 5 min for primer elongation was considered as the optimum, 10, 30 and 40 min hybridisation time were also tested in similar way as described before (Figure S7 c and d).

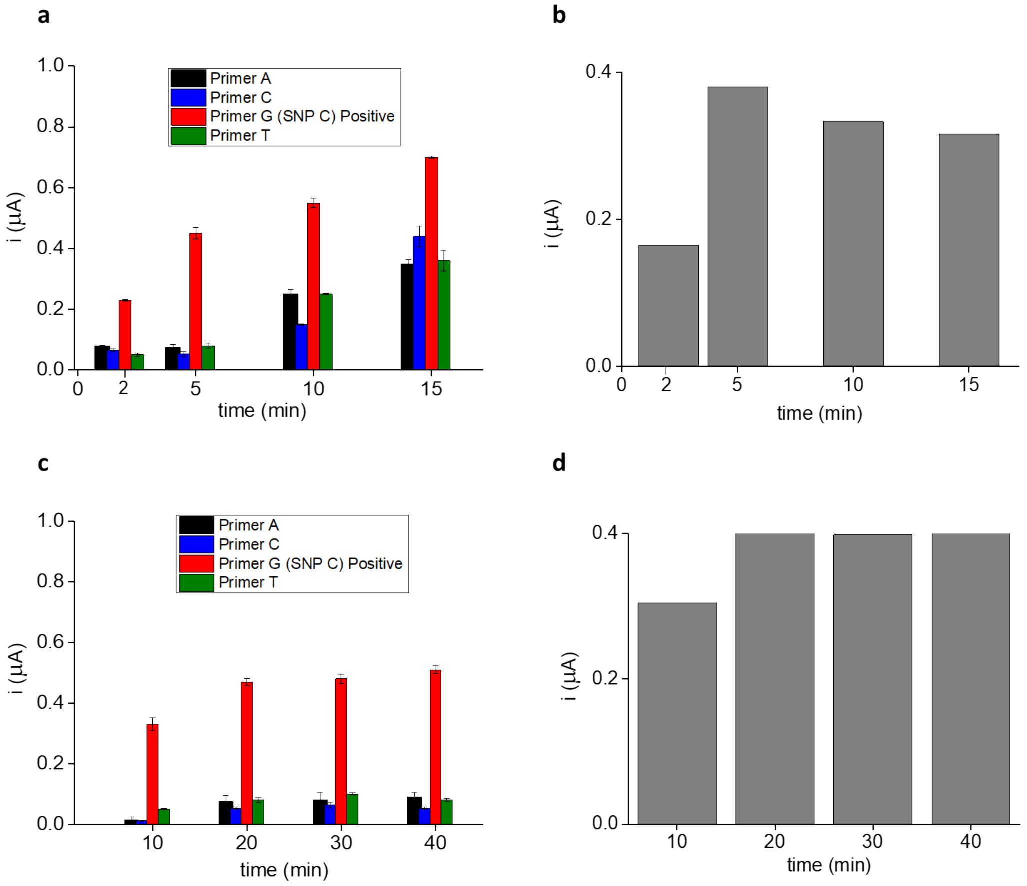

Figure S7. (a) Optimisation of the time of primer elongation reaction after 20 min of hybridisation, based on the SWV signal from the positive signal (Primer G) and three negative controls (non-full complementary primers (A, C and T)). (b) Difference in current intensities between the positive signal (primer G) and the average of the three negative controls (non-full complementary primers A, C and T) vs. reaction time of the primer elongation reaction. (c) Optimisation of the hybridisation time of primer elongation reaction based on the SWV signal from the positive signal (Primer G) and three negative controls (non-fully complementary primers (A, C and T)) followed by 5 min primer elongation. (d) Difference in current intensities between the positive signal (primer G) and the average of the three negative controls (non-full complementary primers A, C and T) vs. hybridisation time.

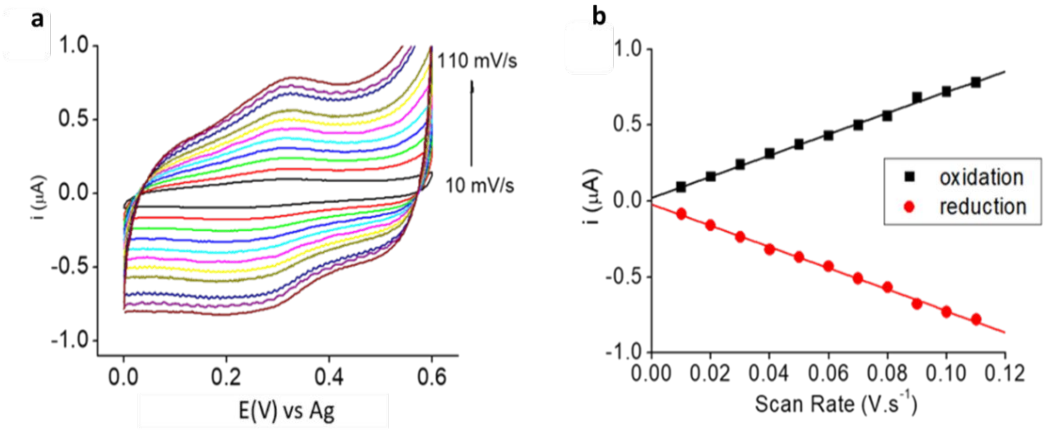

Figure S8. (a) Cyclic voltammograms at different scan rates of surface confined Fc-containing elongation product in 10 mM Tris + 0.1 M KCl. (b) Peak currents vs scan rate plot.

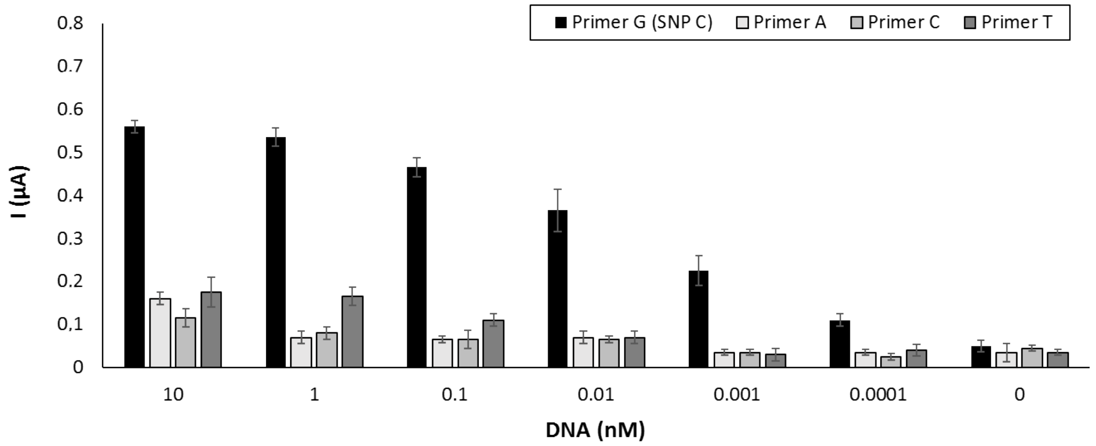

Figure S9. Differential signal over range of DNA concentrations. Signals measured using a range of concentrations of target DNA showing the differentiation between positive signal i.e. primer extension from the immobilised primer with the terminal base complementary to the SNP in the target and negative signals i.e. negligible primer extension from the immobilised primers with terminal bases not complementary to the SNP in the target.

Table S3. Parameters calculated from calibration curves using the best combinations of labeled and natural dNTPs using Sigmoidal four-parameter logistic (4PL) fitting. The LOD indicates the minimum concentration for differential identification of the SNP under interrogation.

**Parameters calculated from calibration curves carried out using the best combinations of labelled and natural dNTPs**					
	**d(A, C, G, U)^{Fc}TP (all bases at 30%)**	**dA^{Fc}TP (100%)**	**dC^{Fc}TP (80%)**	**dG^{Fc}TP (40%)**	**dU^{Fc}TP (50%)**
**Top**	0.440 ± 0.02	0.520 ± 0.04	0.430 ± 0.04	0.480 ± 0.01	0.430 ± 0.05
**Bottom**	0.06 ± 0.05	0.008 ± 0.06	0.020 ± 0.03	0.040 ± 0.02	0.001 ± 0.05
**R^2**	0.9909	0.9923	0.9901	0.9945	0.9978
**LOD (pM)**	3	5	1	0.5	2
**LOD (number of DNA copies)**	27 x10^6	45 x10^6	9 x10^6	5 x10^6	18 x10^6

4.3 Validation of the assay using real samples.

4.3.1 PCR amplification and ssDNA generation.

A common master mix containing 0.2 µM of forward primer and 0.2 µM phosphorylated reverse primer, 1 X KAPA2G Robust PCR buffer, 1 X KAPA2G Robust enhancer, 200 µM of natural dNTPs and 1 unit of the KAPA2G Robust DNA polymerase was prepared and divided in 15 aliquots of 50 µL. Of these, 14 aliquots were used for DNA amplification from 0.5 ng/µL of each of the DNA samples and the last one was used as non-template control.

PCR reactions were carried out in an iCycler Thermal Cycler (Biorad) with the following conditions: 2 min at 95°C, followed by 20 cycles composed by three steps: 1) denaturation during 30 s at 95°C, 2) annealing during 30 s at 58°C and elongation during 30 s at 72°C and a final elongation step for 5 min at 72°C. At the end of the program, the tubes were stored at 4°C.

For ssDNA generation, an asymmetric PCR followed by exonuclease digestion was used. Twenty microlitres of each of the initial PCR reaction were added to 180 µL of master mix containing 0.4 µM of forward primer, 1 X KAPA2G Robust PCR buffer, 1 X KAPA2G Robust enhancer, 200 µM of natural dNTPs and 1 unit of the KAPA2G Robust DNA polymerase. The asymmetric PCR reactions were carried out in an iCycler Thermal Cycler (Biorad) with the following conditions: 2 min at 95°C, followed by 15 cycles composed by three steps: 1) denaturation during 30 s at 95°C, 2) annealing during 30 s at 58°C and elongation during 1 min at 72°C and a final elongation step for 5 min at 72°C. At the end of the program, the tubes were stored at 4°C.

Finally, 2 units of the Lambda exonuclease and 1X Lambda exonuclease buffer were added to the asymmetric PCR products to digest the remaining dsDNA. The reaction was carried out for 2 hours at

37 °C followed by enzyme denaturation at 80 °C for 10 min. At the end of the program, the tubes were stored at 4°C.

Every step was checked by gel electrophoresis as shown in Figure S10. Five microlitres of each sample were mixed with 4 µL of DNA Gel Loading Dye (6X) and spotted in a 2.6 % m/v agarose gel (stained with GelRed nucleic acid stain and prepared in 1X Tris Borate-EDTA buffer (TBE) at pH 8) and the electrophoresis were carried out at 110 V for 30 min in the same buffer.

The samples were purified using Oligo Clean & Concentrator to eliminate remaining primers, enzymes and other reactants. The samples were reconstituted in 15 µL of pure water and the concentration measured using a SimpliNano spectrophotomer.

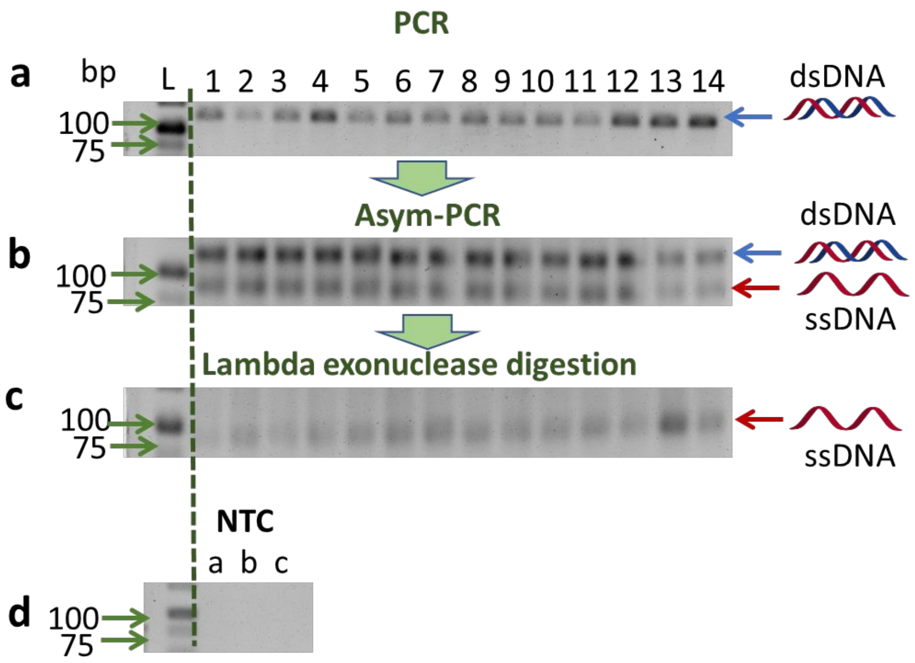

Figure S10. Agarose gel electrophoresis (2.6 % m/v) after different steps of ssDNA generation from DNA extracted from real samples of *Mycobacterium tuberculosis*: a) PCR using forward primer and phosphorylated reverse primer; b) Asymmetric PCR; c) Lambda exonuclease digestion and d) The corresponding NTC of a), b) and c).(L: Ladder, bp: base pairs).

4.3.2 Electrochemical detection of samples

For primer elongation reaction, 15 µL of 0.1 nM of purified ssDNA samples in 10 mM Tris + 500 mM NaCl, pH 7.4 were injected inside the microfluidics and left to hybridise for 20 min at 37 °C to the thiolated reverse primers previously immobilised on an electrode array. After washing with 10 mM Tris Klenow (exo-) DNA polymerase and 200 µM dNTP mixture composed by 30% x (d(A,C,G,U)^{Fc}TP = 7.5% of each dN^{Fc}TP and 70% of unlabelled dNTPs) were injected and left to react at 37 °C for 5 min. After a second washing step, the electrodes were incubated with 0.1 M glycine at pH 3. Finally, the

electrodes were washed twice again with 10 mM Tris + 0.1 M KCl, the channel filled, and the electrochemical measurements carried out using SWV (Figure S11).

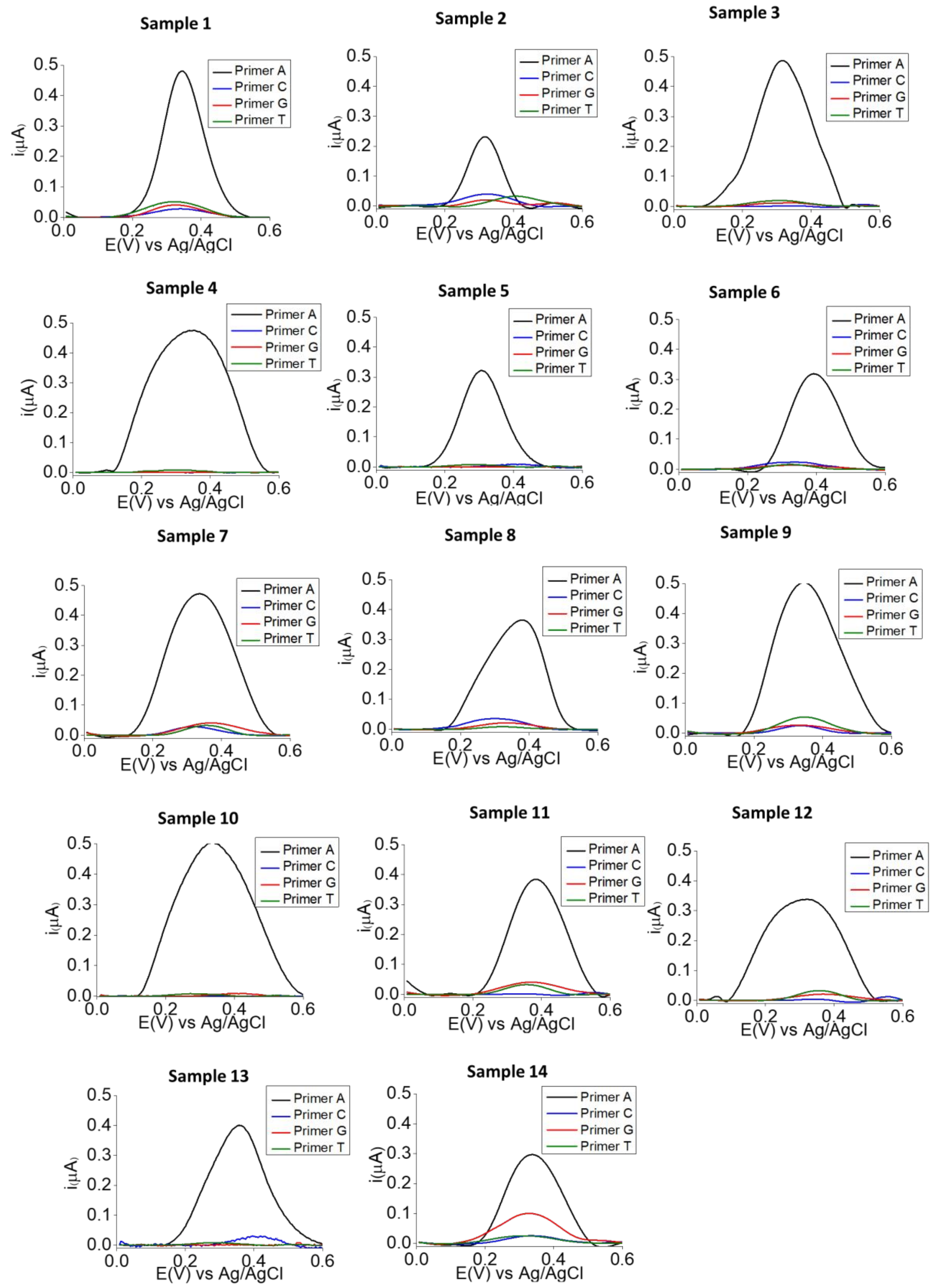

Figure S11. SWVs recorded after solid phase primer elongation reaction using d(A, C, G, U)^{Fc}TP (all bases at 30%) in the reaction mixture of 14 samples containing DNA extracted from *Mycobacterium tuberculosis*.
